# National policies for screening and early detection of breast cancer around the globe: Practices, barriers, and solutions

**DOI:** 10.1002/ijgo.70545

**Published:** 2025-10-01

**Authors:** Versha Pleasant, Charulata Bapaye, Femke Delporte, Maria Jose Del Rio Vigil, Hema Divakar, Gustavo Ferreiro Delgado, Nestor Cesar Garello, Serigne Modou Kane Gueye, Charmaine Clarisse T. Gutierrez, Paola Iturralde‐Roses‐Priego, Christian Jackisch, Sharon Mass, Christine Solbach, the FIGO Committee on Breast Health

**Affiliations:** ^1^ University of Michigan Ann Arbor Michigan USA; ^2^ GEMS Hospital & Endoscopy Centre Pune India; ^3^ Breast Center AZ Sint‐Lucas Brugge Belgium; ^4^ Universidad de Chile Santiago Chile; ^5^ Divakars Specialty Hospital Bengaluru India; ^6^ Gynecology Society of Uruguay Montevideo Uruguay; ^7^ National University of Cordoba Cordoba Argentina; ^8^ Military Hospital of Ouakam University of Ziguinchor Dakar Senegal; ^9^ Calamba Doctors Hospital Laguna Philippines; ^10^ Perpetual Help Medica Center Binan Laguna Philippines; ^11^ Hospital Angeles del Pedregal Mexico City Mexico; ^12^ Evangelische Kliniken Essen Mitte gGmbH Essen Germany; ^13^ Morristown Medical Center Morristown New Jersey USA; ^14^ Breast Center Goethe University Frankfurt Germany

**Keywords:** early detection, health policy, mammography, screening breast cancer

## Abstract

Breast cancer represents a significant burden of disease for women across the globe. Screening has been demonstrated to decrease breast cancer‐related mortality. However, many nations do not have population breast cancer screening programs, which are key to early detection and can decrease mortality rates. In 2024, the International Federation of Gynecology and Obstetrics (FIGO) established the Committee on Breast Health to raise awareness about breast disease, advocate for improved prevention and treatment, and promote best practices. This manuscript aims to review national policies in screening and early detection across 7 of the countries represented in the FIGO Committee on Breast Health: Chile, Germany, India, Mexico, the Philippines, Senegal, and the United States. Policies for population screening for breast cancer are reviewed and compared across countries, as well as efforts in risk stratification. A framework for addressing population screening for breast cancer is also proposed that acknowledges resource and infrastructure limitations across nations. These efforts represent a critical step in addressing breast cancer‐related mortality worldwide.

## 1 INTRODUCTION

Breast cancer represents a significant global burden of disease, with over 2.3 million diagnoses and 670 000 deaths worldwide in 2022 alone.^1^ While some countries with a higher human development index (HDI) have demonstrated a reduction in breast cancer‐related mortality due to increased uptake of screening mammography over decades, breast cancer morbidity and mortality remain elevated in many parts of the world, even within some high HDI countries. According to the World Health Organization (WHO), 1 in 12 women from high HDI countries will be diagnosed with breast cancer and 1 in 71 will die from it. Conversely, in countries with low HDI, 1 in 27 women are diagnosed and 1 in 48 will die from breast cancer.^1^ WHO provides a crude breakdown of 5‐year survival, at approximately 9 in 10 in high‐income countries, 6 in 10 in India, and 4 in 10 in South Africa.^2^

While breast cancer mortality is incredibly complex and can involve factors spanning from awareness to access to treatment, the existence of a national population screening program for breast cancer represents a critical and necessary step for early detection and diagnosis. One systematic review of global breast screening guidelines noted that the majority of guidelines were established in high‐income countries and were similar (recommending annual or biennial screening mammography for average‐risk women aged 40–74 years).^3^ Most of the organized, population‐based screening programs present in 63% of WHO member states were located in high‐income and upper middle‐income countries, many of which were concentrated in Europe (96% of countries). Conversely, only 21% of countries in Africa reported having population screening programs.^4^ Furthermore, data demonstrate that global nations with regular population‐based breast cancer screening programs have 3.74 fewer deaths (95% uncertainty interval [UI], 1.69–5.81) per 100 000 population compared to nations without organized screening programs, with fewer deaths in those aged 50–74 years (10.13 fewer deaths per 100 000; 95% UI, 4.47–15.80). Globally, the existence of an organized population‐based screening program decreases the age‐standardized mortality rate by 1.02% annually (95% UI, 0.71–1.36), compared to a yearly increase of 0.45% in countries with irregular programs (95% UI, 0.23–0.69).^5^

In 2024, the International Federation of Gynecology and Obstetrics (FIGO) established its first Committee on Breast Health to raise awareness of breast disease, advocate for improved prevention and treatment, and promote best practices.^6^ This article reviews the national policies for screening and early detection across seven countries represented across the FIGO Committee on Breast Health: Chile, Germany, India, Mexico, the Philippines, Senegal, and the United States. We review national policies for population screening and risk stratification. Furthermore, we explore a framework for addressing population‐based screening for breast cancer that acknowledges resource and infrastructure limitations within a given country. The objectives of the article are:

1. To review, compare, and contrast national population screening policies for breast cancer across countries.
2. To review other national policies within the scope of screening as well as early detection such as clinical breast exam (CBE), supplemental screening imaging, and breast cancer risk assessment and stratification.
3. To propose a new framework for approaching breast screening in the contexts of low‐, middle‐ and high‐income countries.

### 1.1 National breast cancer population‐based screening policies

Screening mammography has been demonstrated to decrease breast cancer‐related mortality,^7^, ^8^, ^9^, ^10^, ^11^ with screening‐detected lesions showing a survival advantage compared to symptomatic breast cancers.^12^, ^13^ However, there are differences in screening frequency, age of initiation, and modality across countries, as summarized in Table 1 for each country spotlighted in this article.

**TABLE 1:** Screening recommendations by country.

Country	Age of recommended screening initiation	Recommended screening modality	Frequency	Professional society
Chile	Scientific societies/other health actors: 40 years Ministry of Health: 50 years	Mammogram	Age 40–49: Annual (optional) Age 50–69: Annual or biennial Age 70–74: Biennial	Ministry of Health
Germany	50–75 years	Mammography	Biennial	Joint Federal Committee German Society of Senologie German Society of Obstetrics and Gynecology
India	40 years 30 years	Mammogram Clinical breast exam	Annual (Opportunistic) Annual (Recommended)	Ministry of Health and Family Welfare
Mexico	40 years	Mammography	Biennial	Consenso de Colima
Philippines	40 years	Mammography	Annual or Biennial	Philippine Cancer Society
Senegal	40 years	Mammogram	Biennial	Ministry of Health
United States	40‐45 years	Mammography	Annual or biennial	U.S. Preventive Services Task Force^2^ American College of Obstetricians and Gynecologists^1,2^ National Comprehensive Cancer Network^1^ American College of Radiology^1^ American Cancer Society^1,2^

^1^ Annual.

^2^ Biennial.

Established in 1998, the Chilean National Breast Screening Program was initially based on CBE.^14^ While high adherence rates were achieved in Chile (65%) between 2004 and 2007, early‐stage diagnoses of breast cancer were still low.^15^ The Ministry of Health now strongly recommends screening mammography for women aged 50–69 years. With less robust evidence, screening mammography is also recommended for women aged 40–49 and 70–74. Regarding frequency of mammography, it is recommended either annually or biennially. However, there is no established national screening program for which imaging is systematically performed by certified radiologists; thus, the quality of the exams is not always guaranteed.

Across Europe, 32 European Board and College of Obstetrics and Gynecology (EBCOG) member countries are currently providing breast cancer screening programs. While mammography is the universally recommended method for breast cancer screening across EBCOG member countries,^16^ its periodicity varies across countries between annual, biennial, and triennial.^17^ Data from some countries, such as Germany, directly demonstrate the positive impact of a national screening program, showing a mortality reduction of up to 25.8%.^13^ The recommendation in Germany is beinnial mammogram from 50–75 years old.

For some countries, such as India, the overall lack of a national screening program poses challenges to early identification of breast cancer.^18^ The National Cancer Control Programme (NCCP), sponsored by the Ministry of Health and Family Welfare, supports breast screening at primary healthcare centers with a focus on prevention and early detection of cancer.^19^ However, without an established nationwide, population‐based breast cancer screening program, breast screening is largely opportunistic and offered to women aged 40–70 years. Screening services may not always be accessible and uptake remains low.

In Mexico, official regulations previously recommended mammography every 2 years from 40 and 69 years. However, as of July 2023, 30 official Mexican regulations were canceled, which included breast cancer screening and treatment guidelines. Although not a governmental recommendation, the Colima Consensus offers guidelines for the diagnosis and treatment of breast cancer, for which biennial mammography starting at 40 years is now recommended.^20^

The Breast Cancer Control Program (BCCP) established by the Philippine Department of Health supports increased education and treatment of breast cancer,^21^ but faced challenges in implementation and availability of mammography. Screening recommendations from the BCCP emphasized monthly breast self‐examination and annual CBE,^21^ which is still encouraged in low‐resource regions for Filipino women aged 40–69 years. If accessible, screening mammography is recommended every 1–2 years for women aged 40 years and older by the Philippine Cancer Society. However, the lack of an organized breast cancer screening program poses serious challenges in the Philippines.

In Senegal, there is no organized population‐based screening program in place, contributing to low overall screening. This is also within a context of high uncertainty regarding cancer estimates due to the lack of cancer‐based registries, although the Cancer Screening in Five Continents (CanScreen5) data initiative holds promise in better documenting cancer prevalence and screening across the world.^22^ However, recommendations from the Ministry of Health are for biennial screening mammography beginning at 40 years old, for which breast cancer is included in the National Strategic Plan in the Fight Against Cancer.^23^

In the United States, the US Preventive Services Task Force (USPSTF) recommends biennial mammography from 40 and 74 years.^24^ However, there are notable discrepancies across American medical societies regarding recommended age and frequency of screening mammography. The National Comprehensive Cancer Network (NCCN) and American College of Radiology (ACR) recommend annual screening mammography starting at 40 years.^25^, ^26^ The American College of Obstetricians and Gynecologists (ACOG) recommends annual or biennial mammography initiating at 40 years.^27^ The American Cancer Society (ACS) offers the option for women aged 40–44 to begin annual screening mammography, recommends annual mammography from 45 and 54 years, and then recommends annual or biennial mammography for those aged 55 years and older.^28^ Therefore, while a population screening program exists, the discrepancies in guidelines create confusion among primary care practitioners and patients alike, leading to variations in clinical practice.

### 1.2 Supplemental screening guidelines

While mammography is considered the standard of care for average‐risk women, supplemental screening with breast magnetic resonance imaging (MRI) is recommended in many countries for those who are at elevated risk of breast cancer. There is a dearth of data regarding MRI guidelines in countries with low HDI, which could be linked to lack of availability of breast MRI machines and the pre‐existing challenges facing screening for average‐risk communities. Guidelines regarding supplemental screening recommendations for patients at high risk of breast cancer are largely limited to countries with high HDI. In the USA, the NCCN guidelines outline age of initiation and frequency of annual breast MRI screening for high‐risk patients (such as those with germline pathogenic variants such as BRCA, high‐risk breast lesions, increased risk due to family history, or history of chest wall radiation).^29^ In Germany, breast MRI is considered an important component of breast imaging, both for high‐risk screening as well as for diagnostic follow‐up. Recommendations from the Working Group on Breast Diagnostics of the German Radiological Society outline standard techniques for breast MRI utilization.^30^

Breast density is another risk factor for breast cancer that decreases the sensitivity of mammography and for which supplemental screening can improve detection. A multicenter randomized controlled trial based in the Netherlands (DENSE Trial) of MRI screening among women with extremely dense breast tissue demonstrated significantly fewer interval cancers than those undergoing supplemental breast MRI compared to mammography alone.^31^ Despite this benefit and approximately half of the screening population having dense breasts, the guidance for its utilization for this indication remains variable. In the United States, for instance, federal law requires healthcare providers to inform women if they have dense breasts.^32^ However, there is no nationwide consensus regarding screening for women with dense breasts. While other imaging modalities such as breast MRI, whole breast ultrasound,^16^ contrast‐enhanced mammography, and molecular breast imaging have been proposed,^25^, ^26^ supplemental imaging for dense breasts (as a sole risk factor) is not yet universally implemented. Furthermore, ACOG and USPSTF state that there is insufficient evidence for supplemental screening for dense breasts.^24^, ^33^ Patient access to imaging facilities that have the appropriate technology, as well as possible out‐of‐pocket costs, pose barriers.^34^

### 1.3 Clinical breast exam

Most of the countries in this article do not have specific language from professional medical societies regarding the utility of CBE and breast self‐awareness, particularly given the lack of data demonstrating mortality benefit. While CBE is surely warranted in the setting of breast symptoms, the broader data regarding the effectiveness of routine breast exams (by either the healthcare provider or the patient) remain unclear.

In India, annual CBE for women ≥30 years and monthly self‐examination at 20 years are recommended by the Government of India's Expert Consensus Guidelines for Breast Cancer Screening and the Indian Council of Medical Research. The Federation of Obstetric and Gynecological Societies of India (FOGSI) even recommends CBEs for women in their early 20s, with emphasis on awareness of any changes to breast tissue.

In the United States, NCCN recommends clinical encounters every 1–3 years along with ongoing breast self‐awareness initiated at 25 years, and with clinical encounters changing to annually at 40 years. However, it is specified that a “clinical encounter” should include (at a minimum) review of medical and family history with ongoing risk assessment by age 25, risk reduction counseling, and a CBE even in the absence of symptoms.^25^

There is also mention in US‐based NCCN guidelines that CBEs could maximize early detection in low‐resource areas for which routine screening with mammography is limited and cost‐effective options are needed. This is demonstrated in one particular retrospective analysis from Mexico showing that breast cancer was diagnosed in up to two‐thirds of patients through breast self‐examinations.^35^ It is also demonstrated in the Philippines, where breast exams (both self‐examination and clinical examination) are encouraged as screening imaging is limited.^21^

### 1.4 Breast cancer risk assessment and stratification

While the concept of breast cancer screening is most often associated with imaging such as mammography, risk assessment is another form of evaluation to stratify those who may be at high risk, such as those with *BRCA* or other breast cancer susceptibility genes. In the United States, NCCN guidelines largely guide decision‐making regarding those who meet eligibility criteria for genetic testing. NCCN states that all women should undergo breast cancer risk assessment by age 25.^25^ Additionally, the German Consortium for Hereditary Breast and Ovarian Cancer has established multigene panel testing and an interdisciplinary consensus team to better evaluate individual risk and provide clinical recommendations in Germany.^36^ However, risk assessment for breast cancer (involving review of personal risk factors and family cancer history) is not routinely performed across and even within countries with guidelines.

Technologies such as next‐generation DNA sequencing are becoming increasingly available across the world in countries such as Senegal, Chile, Germany, Mexico, India, and the USA. In low‐ and middle‐income countries such as Chile and Mexico, genetic testing remains prohibitively expensive and largely inaccessible to the general population. Despite its availability, this leads to gross underutilization of genetic testing.

## 2 CHALLENGES AND POSSIBLE SOLUTIONS

Breast cancer outcomes are complex and dependent on numerous personal‐, provider‐, system‐, and national‐level factors. Exploring each of these barriers is outside the scope of this article. Rather, in focusing on care at the level of screening, it is evident that the establishment of an organized, national population‐based screening program represents a first and very critical step in establishing breast cancer as a national priority and creating a framework for early detection. WHO provides a rubric of seven elements necessary for the foundation and effectiveness of an organized cancer screening program that includes: documenting policies, ensuring that screening is linked to a pathway for management of positive results, documenting the screening pathway, defining the target audience, inviting the target population to screen based on evidence, ensuring evidence‐based diagnosis and treatment, and implementing quality assurance.^37^

However, we acknowledge that population‐based screening programs are developed within the context of a country's particular infrastructure. For instance, a systematic review of breast cancer screening in sub‐Saharan Africa demonstrates the challenges in care delivery and access, which greatly hinder the implementation of breast cancer screening programs in the region.^38^ It is therefore essential that screening programs be tailored to the resources available, while simultaneously and continuously exploring how improved approaches to early detection in the form of screening technology, risk stratification, and genetic testing can be realized. Table 2 outlines challenges and suggested policy approaches based on HDI level.

**TABLE 2:** Challenges and suggested policy approaches based on human development index.

Human development index	Screening program status	Main technologies	Major challenges	Policy focus
High	Well‐established, population‐based	Mammography, MRI	Discrepancies in guidelines, disparities	Develop population screening program (if one does not exist), Address disparities directly in screening protocols, Incorporate risk stratification, Harmonize recommendations, Emphasize early risk assessment
Middle	Varies; some organized, others opportunistic	Mammography, CBE, Ultrasound	Infrastructure gaps, coverage	Develop population screening program (if one does not exist), Expand infrastructure, Adapt low‐cost methods, Increase utilization of AI, Incorporate risk stratification for personalized screening
Low	Limited/no organized programs	CBE, awareness, symptom‐based, mammography and ultrasound (may be limited)	Resource constraints, infrastructure	Develop population screening program (if one does not exist), Increase international support, Expand infrastructure, Adapt low‐cost methods, Increase utilization of AI, Incorporate risk stratification for personalized screening

Abbreviations: AI, artificial intelligence; CBE, clinical breast exam; MRI, magnetic resonance imaging.

While there are established population‐based screening programs in many high HDI countries, there may be inconsistencies in messaging across medical societies regarding the initiation and frequency of screening. There should be improved harmonization of messaging for average‐risk screening and for supplemental screening for dense breast tissue, along with improved identification of those at increased risk through risk assessment by age 25 and genetic testing of those who meet the criteria. There is also a need to address those subpopulations within high HDI counties with higher breast cancer mortality. For instance, in the USA, although black women are almost just as likely to be diagnosed with breast cancer, they have a 38% increased risk of breast cancer mortality.^39^ It is important that policies are tailored to appropriately address this risk with the goal of reducing disparities.

For middle‐ and low‐HDI countries, infrastructure gaps may pose challenges to establishing an organized screening program. While these resources are being explored and expanded (for which the mainstay would be procurement and accessibility of screening mammography), other low‐cost methods could be explored. For instance, while CBE does not decrease breast cancer mortality, it is low cost, widely available, and could represent a possible screening method in generally unscreened communities.^40^ One particular study from Mumbai demonstrated a 15% nonsignificant overall reduction in mortality and a significant 30% reduction in mortality (for women aged over 50 years) with biennial CBE by a healthcare provider, as well as significantly downstaged breast cancers at diagnosis.^41^ An overview of systematic reviews examining CBE as a standalone screening tool in low‐ and middle‐income countries did not show mortality benefit, but indirectly suggested that thorough CBE could mirror a similar effect as mammography with lower sensitivity (40%–69% for CBE vs. 77%–95% for mammography).^42^ The overview also demonstrated a 17%–47% shift from advanced to early‐stage cancer with CBE.^42^ This suggests the potential role that CBE could play in low‐resource settings in which mammography may not be available.

Breast ultrasound also represents a possible option in low‐resource settings, although this remains controversial as a form of primary screening. It represents noninvasive, radiation‐free imaging that carries lower costs than other imaging modalities. One Chinese prospective study evaluated the utility of automated breast ultrasound with remote reading for primary breast screening. The cancer detection rate was 4.0 per 1000 women, with a sensitivity of 92.3% (95% CI, 75.9%–97.9%) and specificity of 88.4% (95% CI, 87.6%–89.2%), which met the benchmark performance for cancer detection when compared to mammography.^43^ A retrospective study in China also showed higher sensitivity of ultrasound than mammography, especially in patients with dense and smaller breasts.^44^

Personalized screening plans continue to be explored as a tool for breast cancer risk stratification and should be considered for population‐based screening in limited settings. One systematic review examining cost‐effectiveness in low‐ and middle‐income countries demonstrated that mammography generates greater costs and requires training for utilization, but is cost‐effective and could be adapted based on country context for age and risk factors.^45^ A cost‐effectiveness study was performed in the United Kingdom's National Breast Cancer Screening Program using an artificial intelligence (AI) risk‐prediction model to inform mammography screening intervals. This model assigned mammography every 6 years for those at low risk, every 2–3 years for those at below or above‐average risk, and annually for those at highest risk. This decision analytical model suggested some cost benefit to risk stratification, with estimated quality adjusted life year value of £1 (US $1.28) to generate a yearly monetary benefit of an estimated £10.6 million (US $13.6 million).^46^

Aside from cost savings, measuring the effectiveness of such an approach is also critical. MyPeBS (My Personal Breast Screening) is an ongoing randomized controlled trial funded by the European Union (involving Italy, France, Israel, the UK, Belgium, and Spain) exploring personalized breast cancer screening.^47^ The study includes 85 000 women aged 40–70 randomized to standard screening versus risk evaluation, for which they are stratified into low, moderate, high, or very high risk. These categories subsequently inform the frequency and modality of breast cancer screening (i.e. moderate risk women receive mammography every 2 years versus high risk women who receive annual mammography). Similarly, breast screening guidelines set forth by the Ministry of Health in Malaysia are a real‐life example of population screening based on a risk‐stratified approach. CBE and mammography are recommended but are also offered opportunistically. However, the Ministry of Health's Management of Breast Cancer Clinical Practice Guidelines involves breast cancer risk assessment that factors in family cancer history and the presence of hereditary cancer syndromes to inform the age of initiation, frequency, and type of breast cancer screening.^48^ While data are still forthcoming on the effectiveness of these models, this approach could potentially offer a more sustainable solution, especially in low‐resource settings for which population‐level annual screening mammography may not yet be feasible.

AI can also be leveraged to analyze imaging results quickly and accurately, aiding in the early detection of breast cancer and reducing the burden on radiologists in resource‐limited settings. AI can act as a second reader, providing an independent assessment of mammograms and helping to reduce false‐positives or missed diagnoses. Even in high‐resource settings, AI can help detect subtle patterns and anomalies that might be missed by the human eye, even by experienced radiologists. MASAI, a randomized controlled trial using AI‐supported screen reading, demonstrated similar breast cancer detection rates (compared with standard double screening) and decreased screening workload.^49^ Another Swedish‐based prospective, population‐based study (ScreenTrustCAD) demonstrated a 4% higher noninferior breast cancer detection rate using AI compared with a radiologist double reading.^50^ The Artificial Intelligence for Breast Cancer Screening in Mammography (AI‐STREAM) is a Korean‐based prospective, multicenter study comparing the diagnostic accuracy using a computer‐based detection tool in mammography readings, with findings demonstrating increased breast cancer detection rate of 13.8% for radiologists using computer‐aided detection (*n* = 140 [5.70‰]) compared with those without AI (*n* = 123 [5.01‰]; *P* < 0.001).^51^

Finally, international aid organizations that aim to invest in developing long‐term and sustainable health systems in middle‐ and low‐HDI countries must begin to consider the burden of not just infectious diseases, but noncommunicable diseases that also impact mortality. There is incredible potential for capacity‐building in this sector, creating synergies with nations prioritizing breast cancer screening policies who may need additional infrastructure and resource support. To address the global burden of breast cancer, WHO launched the Global Breast Cancer Initiative (GBCI) in 2021, which involves three primary strategies: health promotion and early detection, timely diagnosis, and comprehensive breast cancer management. The goal of the GBCI is to reduce global breast cancer‐related mortality by 2.5% by the year 2040, which will save an estimated 2.5 million lives.^52^

## 3 CONCLUSION

As screening is key to addressing breast cancer mortality across the globe, the establishment of organized, national, population‐based screening programs is a critical component of this effort. We simultaneously recognize the limitations in low‐resource settings, for which we anticipate that individualized, risk‐based programs increasingly supported by AI (for both risk stratification to inform screening intervals and modality, as well as to assist in imaging interpretation) may occupy a more prominent role and create more effective interim solutions. The role of CBE must be considered, along with increasing research into other low‐cost screening methods. Gathering family cancer history is a simple, low‐cost tool to aid in breast cancer risk stratification and can be incorporated into population‐based screening policies. Genetic testing is the foundation of precision medicine and must be prioritized to reach all global communities, further aiding in risk stratification. Furthermore, while we recognize the competing priorities of international aid organizations in addressing the urgency of communicable diseases, we implore the global community to consider expanding its scope of care to include noncommunicable pathologies, such as breast cancer, that carry significant morbidity and mortality. Breast cancer screening policies must be prioritized to create more sustainable, meaningful long‐term care to women across the world.

## AUTHOR CONTRIBUTIONS

All authors contributed to generating the concept for the manuscript, research, writing, revising and approval of the final version.

## FUNDING INFORMATION

We have not received any specific funding support to conduct this study.

## CONFLICT OF INTEREST STATEMENT

Versha Pleasant is the owner of Pleasant Consulting LLC, which provides lectures and talks on cancer genetics, breast cancer‐related health disparities, and gynecologic care of breast cancer survivors and high‐risk patients. There are no conflicts of interest among the remaining authors.

## Data Availability

Data sharing is not applicable to this article as no new data were created or analyzed in this study.
